# Assessment of Mitochondrial Function and Oxygen Consumption Measured During *Ex Vivo* Normothermic Machine Perfusion of Injured Pig Kidneys Helps to Monitor Organ Viability

**DOI:** 10.3389/ti.2022.10420

**Published:** 2022-05-31

**Authors:** James P. Hunter, Letizia Lo Faro, Kaithlyn Rozenberg, Fungai Dengu, Anne Ogbemudia, Annemarie Weissenbacher, John F. Mulvey, Laura Knijff, Kishore Gopalakrishnan, Rutger J. Ploeg

**Affiliations:** ^1^ Nuffield Department of Surgical Sciences, Medical Sciences Division, University of Oxford, Oxford, United Kingdom; ^2^ University Hospitals Coventry and Warwickshire NHS Trust, Coventry, United Kingdom; ^3^ Department of Visceral, Transplantation and Thoracic Surgery, Medical University of Innsbruck, Innsbruck, Austria; ^4^ Leiden University Medical Center, Leiden, Netherlands

**Keywords:** kidney, mitochondria, normothermic machine perfusion, ischemia/reperfusion injury, preservation

## Abstract

Donor kidney assessment may improve organ utilisation. Normothermic Machine Perfusion (NMP) has the potential to facilitate this advance. The mechanism of action is not yet determined and we aimed to assess mitochondrial function during NMP. Anaesthetised pigs (*n* = 6) had one kidney clamped for 60 min. The healthy contralateral kidney was removed and underwent NMP for 8 h (healthy control (HC), *n* = 6). Following 60 min warm ischaemia the injured kidney underwent HMP for 24 h, followed by NMP for 8 h (*n* = 6). Mitochondria were extracted from fresh tissue for analysis. Injured kidneys were analysed as two separate groups (IMa, *n* = 3 and IMb, *n* = 3). Renal resistance was higher (0.39ï, ± 0.29 vs. 1.65ï, ± 0.85; *p* = 0.01) and flow was lower (55ï, ± 28 vs. 7ï, ± 4; *p* = 0.03) during HMP in IMb than IMa. NMP blood flow was higher in IMa versus IMb (2-way ANOVA; *p* < 0.001) After 60 min NMP, O_2_ consumption was significantly lower in IMb versus IMa (*p* ≤ 0.002). State-3 respiration was significantly different between the groups (37ï, ± 19 vs. 24ï, ± 14 vs. 10ï, ± 8; nmolO_2_/min/mg; *p* = 0.049). Lactate levels were significantly lower in IMa versus IMb (*p* = 0.028). Mitochondrial respiration levels during NMP may be suggestive of kidney viability. Oxygen consumption, renal blood flow and lactate can differentiate severity of kidney injury during NMP.

## GRAPHICAL ABSTRACT

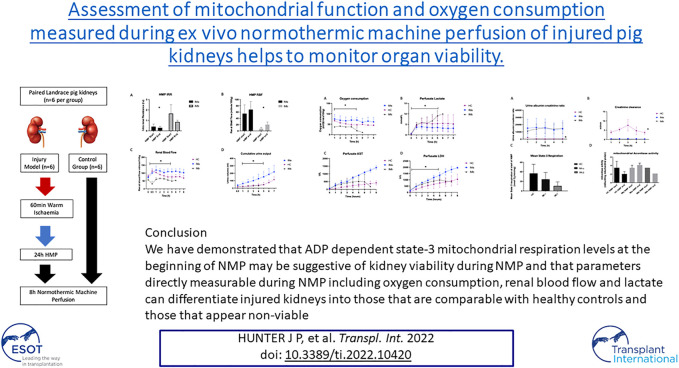

## Introduction

The population of organ donors has shifted in recent decades from younger healthy patients with isolated cerebral trauma to older and higher risk patients with more co-morbidities. The result is that clinicians accepting organs for transplant have increasing uncertainty about organ quality. The ability to accurately predict the quality of an organ by a safe and reproducible assessment prior to transplant is desirable for both patients and clinicians. As a result of the drive to utilise more organs there has been a steady increase in the use of donation after circulatory death (DCD) kidneys [1], which sustain warm ischaemic injury. Following transplantation this injury is amplified and results in ischaemia-reperfusion injury (IRI) which is a complex cascade involving immune mediation, mitochondrial dysfunction, complement activation and oxidative damage to cells and tissues [2]. Attempts to minimise IRI by improving organ preservation methods may improve outcomes following transplant.

Kidney normothermic machine perfusion (NMP) is a preservation method that is based on pumping an oxygenated red blood cell-based perfusion solution *via* the renal artery at physiological temperature. NMP reanimates the organ and allows the measurement of perfusion parameters such as renal blood flow and renal resistance, metabolic parameters such as pH and acid-base balance and measures of function such as creatinine clearance. NMP also offers the opportunity to assess organ viability and in future may provide enough information for clinicians to base decisions on whether to accept an organ for transplant [3]. Cerebral injury followed by organ preservation with standard cold preservation causes hypoxia with a significant stunning of mitochondria and cellular energy metabolism whilst adequate function after transplantation of the kidney depends on immediate cellular ATP production. Short periods of NMP can replenish ATP and may improve early outcomes after transplant [4]. Recently published work showed that prolonged NMP of discarded human kidneys was feasible and there was some evidence of histological improvement at the end of perfusion [5]. However, the mechanisms of action of NMP are not clear and to target interventions or improve means of assessment a better understanding is necessary.

The impact of IRI on mitochondrial function is significant and has direct and indirect consequences. Unrecoverable mitochondrial injury leads to mitophagy and loss of mitochondria which has a direct impact on cell energetics and may lead to apoptosis/necrosis [6]. Indirect consequences of mitochondrial injury result from the production of free radicals which cause damage to DNA, proteins, cell organelles and lipids [7]. To date, assessment of mitochondrial function during hypothermic oxygenated machine perfusion (HOPE) and NMP has been performed in rat and clinical models of liver transplant, but not in kidneys [8]. Given the pivotal role of mitochondria as the cellular engine we hypothesised in this study that NMP would aid mitochondrial recovery in a pig model of kidney injury.

## Materials and Methods

### Animals and Materials

Animal welfare and experimental procedures were in adherence with Home Office codes of practice and the Animals (Scientific Procedures) Act 1986. Study design was ratified and local ethical approval was obtained through standard University of Oxford procedures. Landrace pigs (50–60 kg) were used.

### Sample Size Calculation

Sample size was based on Mitochondrial oxygen consumption, ATP levels and Complex I activity and calculated from previous rat kidney studies. The calculations yielded groups sizes of 3, 7, and 8 respectively and following discussion with our local animal ethics board, *n* = 6 was selected to balance the principle of Reduction with statistical power. Full power calculations are detailed in Supplementary Appendix SA2.

### Study Design

**FIGURE 1:**
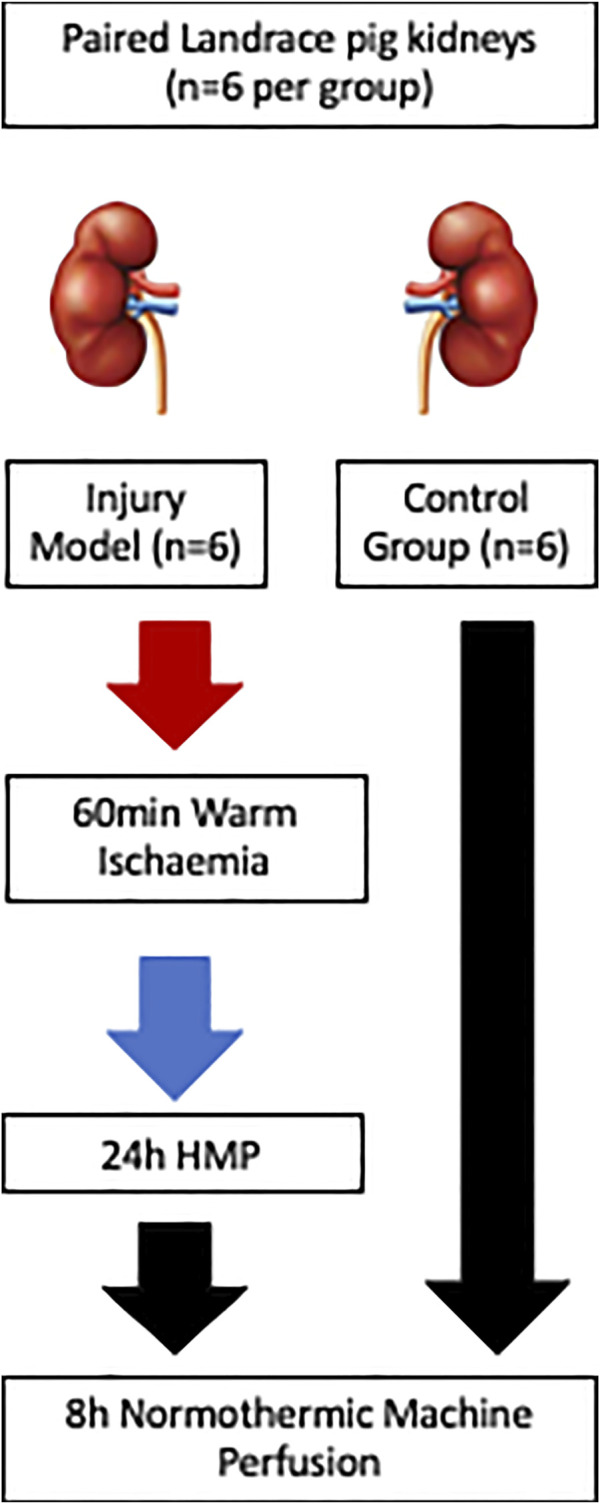
Schematic diagram of the study methods illustrating the differing injury sustained and preservation methods between the injury groups (*n* = 6) and the control group (*n* = 6).

Paired kidneys from pigs (*n* = 6) were allocated to either uninjured control or injury group. Healthy controls (HC) were retrieved, static cold stored (3 h) during transfer back to the laboratory and perfusate preparation, and placed on normothermic machine perfusion (NMP) for 8 h. The injury model group kidneys (IM) were subjected to 60 min warm ischaemia followed by 24 h hypothermic machine perfusion (HMP) and then 8 h NMP, Figure 1. The combination of warm and cold ischaemia was used to simulate the injury sustained in DCD donation. The degree of injury was selected following pilot pig auto-transplants performed by collaborators in the MePEP consortium, a collaboration between University of Aarhus, Erasmus University Rotterdam, University Medical Centre Groningen and University of Oxford funded by the Lundbeck Foundation. The experiments demonstrated that 75 min warm ischaemia followed by 16 h cold ischaemia (CI) and auto-transplant caused significant acute kidney injury but no mortality [9]. For logistical reasons 16 h CI was not practical so to account for the increase in CI to 24 h, we reduced the WI from 75 to 60 min.

### Anaesthesia

Animals were sedated and anaesthetized following standard protocols for the study centre. Anaesthesia was maintained with isoflurane in oxygen *via* positive-pressure ventilation. Remifentanil 2 μg/ml (GlaxoSmithKline) was given as a continuous intravenous infusion for analgesia, and maintenance fluids were delivered at 2 ml per kg per h (Hartmann’s solution, Aqupharm; Animalcare, York, United Kingdom) throughout surgery. Oxygen saturation, heart rate, respiratory rate, expired carbon dioxide level, body temperature, electrocardiography and blood metabolic parameters were monitored throughout surgery. 25,000 units heparin was given intravenously prior to clamping of the renal vessels.

### Surgical Technique

The pig was placed supine and the abdomen opened through a midline incision. The kidney allocated to the injury group was dissected and the renal artery and vein were exposed and cross-clamped for 60 min. During the period of warm renal ischaemia, the contralateral kidney, allocated to healthy control, was dissected free from surrounding attachments. 10 min prior to the end of 60 min WI the healthy kidney was removed, flushed with 250 ml Soltran and placed on static cold storage (SCS). At the end of 60 min WI the kidney was removed, flushed with 250 ml Soltran and placed on HMP.

### Hypothermic Machine Perfusion

The renal artery was cannulated and the kidney was connected to the Kidney Assist-transport device (Kidney Assist transport, XVIVO. Groningen, Netherlands). The circuit was filled with 500 ml of cold Belzer MPS^®^ UW solution (Bridge to Life Ltd., United Kingdom). Perfusion was commenced at an arterial pressure of 25 mmHg and renal blood flow, renal resistance and temperature were monitored throughout perfusion.

### Normothermic Machine Perfusion

The Kidney Assist device (Kidney Assist, XVIVO, Groningen, Netherlands) was used for NMP. Kidney Assist consumables were used however the circuit was modified to minimise the length of tubing and to accommodate a custom organ chamber. The device was primed with perfusate containing albumin and an autologous red blood cell (RBC) suspension added to produce a haematocrit of 25%. Following cannulation of the renal artery the kidney was connected and perfusion was commenced at a pressure of 70 mmHg. Urine volume was replaced with Ringer’s lactate diluted with sterile water to half strength to maintain electrolyte homeostasis and avoid hypernatraemia.

### Perfusate

Autologous red blood cells (RBC) were prepared from whole blood. Blood was filtered using a leukocyte filter (CompoFlex, Fresenius Kabi, Bad Homburg vor der Hohe, Germany) and then centrifuged at 3,000 x g for 20 min at room temperature. The RBCs were washed with 1x phosphate-buffered saline (PBS) and removed by centrifugation at 3,000 x g for 20 min. The washed, isolated RBCs were used for perfusion and added to obtain a haematocrit of 25%. The perfusion solution contained: 250 ml 5% human serum albumin (Alburex, CSL Behring UK Limited, West Sussex, RH16 1AH, UK); 6 ml glucose (B. Braun); 3 ml calcium gluconate (B. Braun); 10 mg mannitol (Sigma Aldrich) and 1000 μmol/L creatinine (Sigma Aldrich). The pH was buffered with 5 ml 8.4% sodium bicarbonate to a physiological pH of 7.2–7.45. The perfusate was supplemented with 1200 mg Co-amoxiclav. A Verapamil bolus of 0.25 mg was added at the beginning of NMP and then 0.25 mg/h was added. Prior to commencing NMP, perfusate was analysed using ABL90 FLEX blood gas analyser to ensure values were physiological. Any alterations to normalise electrolyte and acid-base balance were made prior to connecting the kidney and commencing perfusion.

### Sampling

Perfusion characteristics including renal blood flow, intrarenal resistance and urine production were monitored. Arterial perfusate, venous perfusate and urine samples were taken hourly and blood gas analysis was carried out hourly on the ABL90 FLEX blood gas analyser (Radiometer, Copenhagen, Denmark). Blood and urine samples were centrifuged at 12,000 x g for 12 min at 4°C and the supernatant stored at -80°C for further analysis. Punch and tru-cut needle biopsies were taken prior to NMP after 1 h NMP and at the end of perfusion. Early analysis of mitochondrial function in the injured kidney group suggested evidence of recovery and so additional biopsies were taken at 2, 4 and 6 h in the final 3 kidneys. Biopsies were either prepared immediately to extract mitochondria, stored in formalin or snap-frozen in liquid nitrogen and stored at −80°C.

### Wet/Dry Ratio

Punch biopsies (5 × 8mm) were collected, weighed and dehydrated for 24 h in an incubator set at 60°C. They were then re-weighed and the wet/dry (W/D) weight ratio was calculated by dividing the wet by the dry weight. This ratio determines the proportion of accumulated fluid during the perfusion and the greater the ratio, the greater the volume of fluid accumulated.

### Mitochondrial Assessment

#### Mitochondrial Isolation and Respiration

Mitochondria were isolated from the biopsies collected during perfusion and their function was assessed by O_2_ consumption in a Clarke-type electrode, as previously described [10]. Briefly, biopsies were placed on a Petri dish on wet ice in 1:10 (weight: volume) ice-cold mitochondria isolation buffer (IBc, Tris-MOPS 10 mM, EGTA/Tris 1mM, Sucrose 0.2M, pH 7.4). Biopsies were minced with a scalpel and subsequently homogenised in a pre-cooled glass-teflon homogeniser. Homogenates were centrifuged at 600 x g for 10 min at 4°C. The supernatants were transferred to pre-cooled clean tubes and further centrifuged at 7,000 x g for 10 min at 4°C. The supernatant was then discarded and the pellet containing the mitochondria was gently suspended by pipetting. 25 uL of the mitochondrial suspension were then added to an oxygen electrode (Oxygraph, Hansatech) chamber, equilibrated at 30°C with 1 ml of EBc buffer (KCl 0.125M, Tris/MOPS 10 mM, EGTA/Tris 10 mM, P_i_ 1 mM). Oxygen concentrations (nmol/ml) were recorded. Using Hamilton micro syringes, succinate (final concentration in chamber 5 mM) was added to the chamber and state 2 respiration O_2_ consumption was recorded. After 5 min, ADP was injected into the chamber (final concentration 150 µM) and the state 3, ADP-dependent mitochondrial respiration was recorded. Mitochondrial O_2_ consumption following the addition of ADP was calculated and normalised to mitochondrial protein concentration in each sample. Mitochondrial protein concentration was determined by BCA assay (Thermo Scientific) according to manufacturer’s instructions.

### Mitochondrial Aconitase Activity Assay

Aconitases are a family of iron-sulfur enzymes that catalyse the conversion of citrate to isocitrate. Aconitases are reversibly inactivated by reactive oxygen species, and aconitase activity in the mitochondrial compartment is thus indicative of mitochondrial oxidative stress. Aconitase activity in the mitochondria isolated from the kidney biopsies collected before and at the end of NMP was assessed by a colorimetric aconitase activity assay (Sigma Aldrich), per manufacturer’s instructions. Mitochondrial aconitase activity in these samples was normalised to the mitochondrial protein concentration.

### Histology

Samples were preserved in formalin, processed, sliced and stained using Haematoxylin and Eosin. Tissue injury was assessed using four categories; tubular dilatation, tubular sloughing, cytoplasmic vacuolation and apoptosis. For each category, a severity of injury was ascribed from 0–3; 0 = normal, 1 = mild, 2 = moderate and 3 = severe. The sum of the scores were calculated for each kidney biopsy at three time points (T0 = prior to perfusion, T1 = after 1 h NMP and Tend = end of NMP). Depending upon the sum of the scores, kidneys were then allocated to one of three groups of injury for each time point: A = 0–2, B = 3-4 and C =>5. Slides were assessed by a renal pathologist who was blinded to the grouping (HC or IM).

### Statistical Analysis

Data are presented as mean (s.d.) or as median (range). Normality was assessed using the Shapiro-Wilk test, and non-parametric data were analysed with the Mann–Whitney *U* test. Continuous variables were analysed using Area under the curve (AUC) and One or two-way analysis of variance (ANOVA) and multiple groups were compared using one or two-way analysis of variance (ANOVA) with Tukey’s multiple comparisons. Categorical data were compared using Chi-squared test. Statistical analysis was performed using InStat and Prism 8 and 9 software (GraphPad Software, San Diego, CA, United States). *p <* 0.050 was considered statistically significant.

## Results

One kidney allocated to the injury group was hydronephrotic and anatomically abnormal and was excluded. To replace this excluded kidney and ensure *n* = 6 in each group an additional experiment was performed. Three kidneys in the injury model (IM) group had perfusion stopped prior to 8 h due to macroscopic appearance (globally poor perfusion) and according to the criteria for perfusion termination in Supplementary Appendix SA1. Kidneys in the IM group were either able to perfuse successfully for 8 h (*n* = 3) or were terminated early (*n* = 3). As a result, we have analysed the IM kidneys in two groups, those that perfused successfully for 8 h (IMa) and those that were terminated early (IMb).

### Weight and Wet/Dry Ratio

There was no significant change in weight during NMP in any of the groups, although HC and IMa kidneys had a mean increase in weight of 20% and 10%, respectively and IMb kidneys had a mean reduction in weight of 12% (data missing for final IMb kidney, therefore *n* = 2 so unable to perform any statistics).

### Hypothermic Machine Perfusion

**FIGURE 2:**
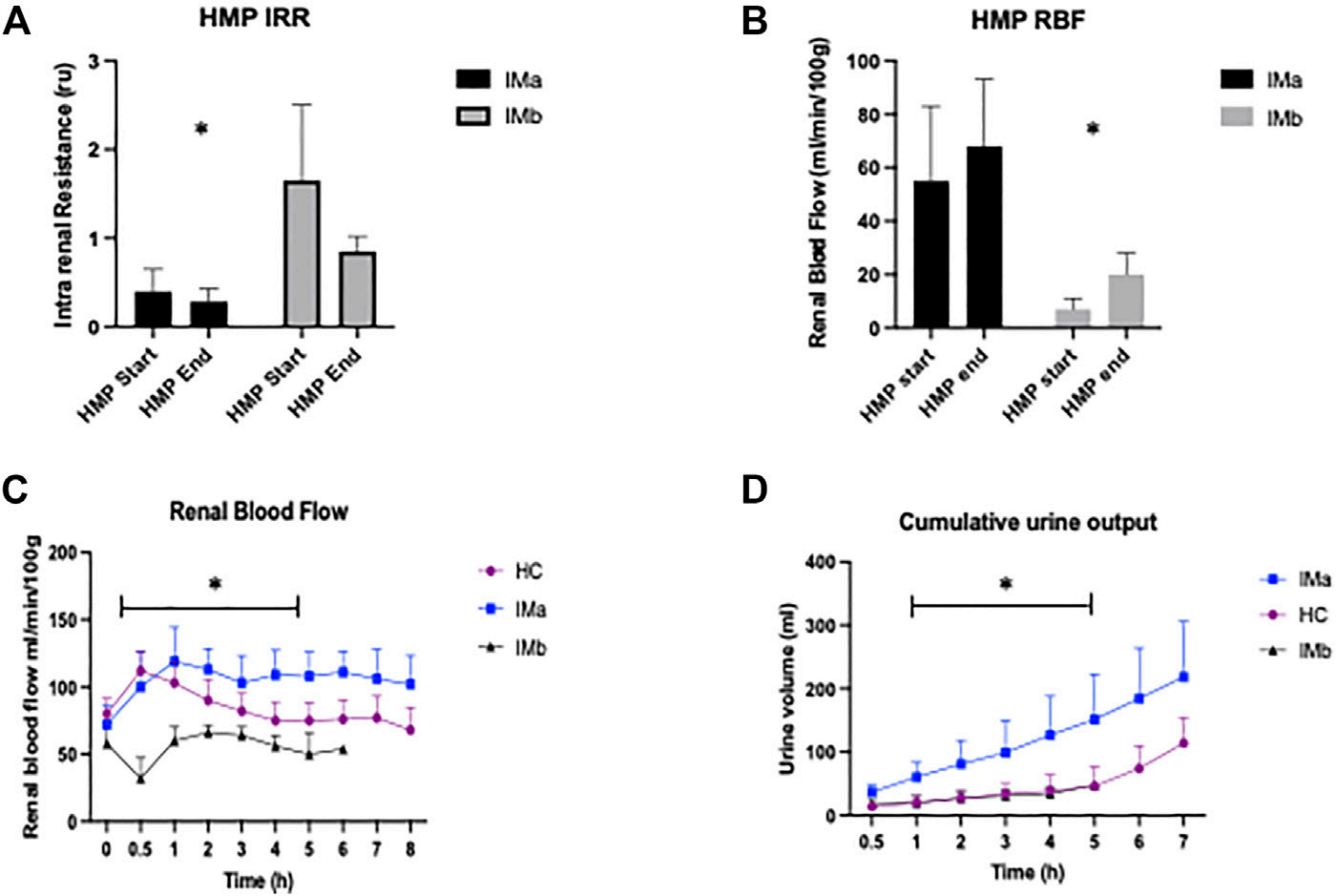
**(A)** Histogram showing Intrarenal resistance (IRR) and **(B)** Renal blood flow (RBF) during Hypothermic Machine Perfusion (HMP) of Injury model kidney groups IMa and IMb (1-way ANOVA with = *p* < 0.05 *). **(C)** Line graph showing renal blood flow and **(D)** Cumulative Urine Output during Normothermic Machine Perfusion of Healthy and Injury model kidney groups IMa and IMb (2-way ANOVA analysed over 5 h NMP with = *p* < 0.05*).

Intrarenal resistance decreased in both IM groups and there was a significantly lower mean intrarenal resistance at the start of HMP (T0) between IMa and IMb (2-way ANOVA: 0.39 ± 0.29 vs. 1.65 ± 0.85; *p* = 0.01) but not at the end of HMP (2-way ANOVA: 0.25 ± 0.14 vs. 0.51 ± 0.17; *p* = 0.76), Figure 2. Renal blood flow increased throughout HMP in both groups and was significantly higher at the start (2-way ANOVA: 55 ± 28 vs. 7 ± 4; *p* = 0.03) and end (2-way ANOVA: 68 ± 25 vs. 20 ± 8; *p* = 0.03) of HMP in the IMa compared to IMb group, Figure 2.

### Normothermic Machine Perfusion

#### Renal Blood Flow

There was a significant difference in RBF between the three groups (2-way ANOVA: *p*=<0.001). RBF was significantly higher in HC compared with IMb (2-way ANOVA with Tukey’s multiple comparisons; *p* = 0.029) and IMa compared with IMb (2-way ANOVA with Tukey’s multiple comparisons; *p* < 0.001) and interestingly RBF was higher in IMa compared with HC (2-way ANOVA with Tukey’s multiple comparisons; *p* = 0.002), Figure 2. The groups, including Tukey’s multiple comparisons were compared over 5 h NMP, which was the duration of perfusion with all 12 kidneys included.

### Cumulative Urine Production

There was a significant difference in cumulative urine production between the three groups (2-way ANOVA: *p*=<0.0029). Urine production was significantly higher in HC and IMa when compared with IMb (2-way ANOVA with Tukey’s multiple comparisons; *p* = 0.005 and *p* = 0.004, respectively). There was no difference in urine output between HC and IMa (2-way ANOVA with Tukey’s multiple comparisons; *p* = 0.8). The groups including Tukey’s multiple comparisons were compared over 5 h NMP, which was the duration of perfusion with all 12 kidneys included, Figure 2.

### Oxygen Consumption

**FIGURE 3:**
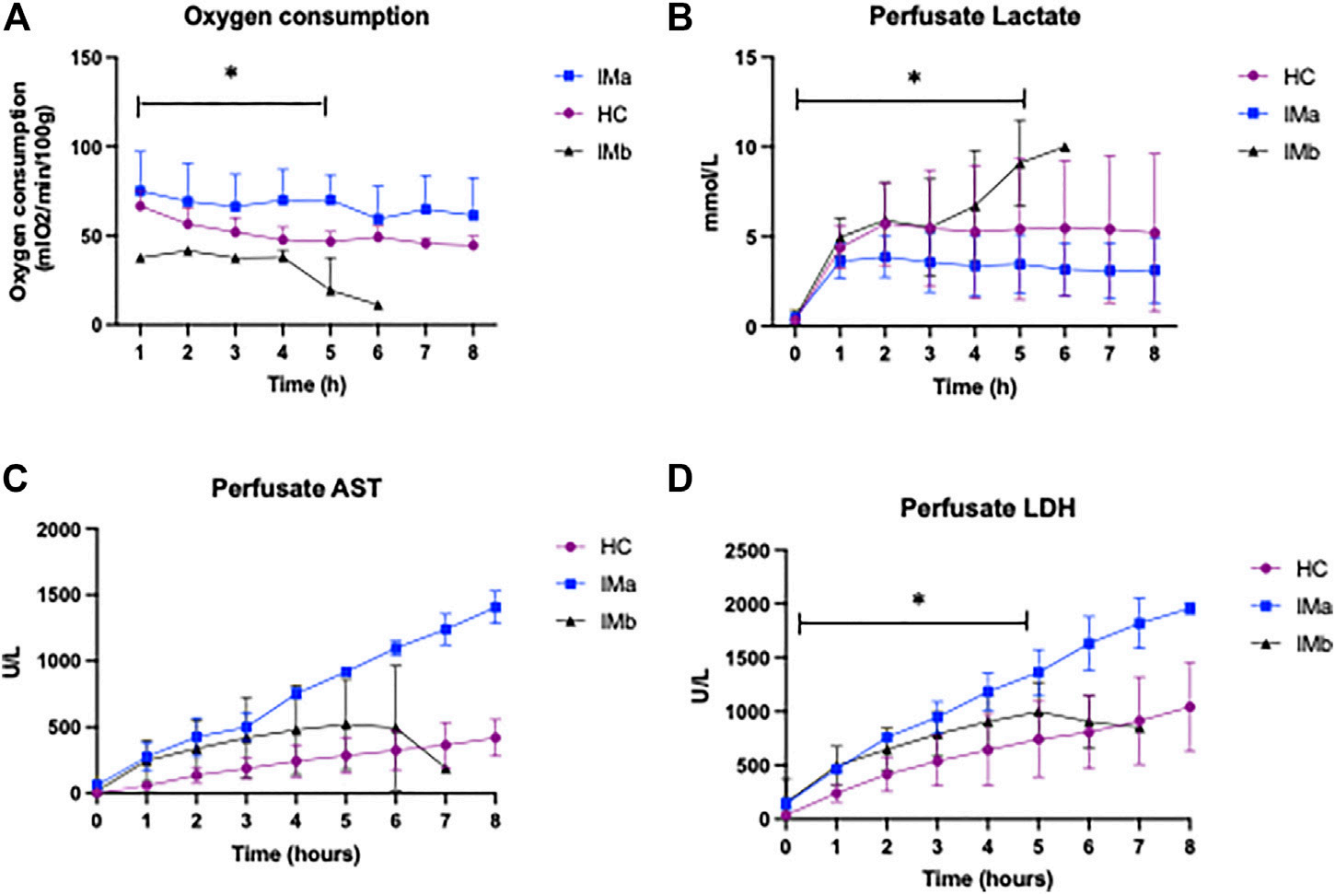
**(A)** Line graph showing oxygen consumption **(B)** Perfusate lactate levels **(C)** Perfusate AST and **(D)** Perfusate LDH levels during Normothermic Machine Perfusion of Healthy and Injury model kidney groups IMa and IMb (2-way ANOVA analysed over 5 h NMP with = *p* < 0.05 *). AST, aspartate transaminase, LDH, lactate dehydrogenase (* = *p* < 0.05).

There was a significant difference in O_2_ consumption between the three groups over 5 h NMP (2-way ANOVA *p* = <0.0001). O_2_ consumption was significantly higher in IMa compared with IMb and HC (2-way ANOVA with Tukey’s multiple comparisons *p* = 0.002 and *p* = 0.04, respectively) and HC compared with IMb (2-way ANOVA with Tukey’s multiple comparisons over 5 h NMP, *p* = 0.04), Figure 3. Two of the three ischaemic kidneys that were terminated early, at hours 5 and 6 showed an inability to consume oxygen with PvO_2_ increasing from 9 to 76 kPa and 16–50 kPa over the final hour and with oxygen consumptions of 1 and 11 kPa/ml/min/g respectively. The median PvO_2_ and oxygen consumption of the 3 injured kidneys that completed 8 h NMP was 8.6 kPa and 57 kPa/ml/min/g, respectively.

### Lactate

There was a significant difference in perfusate lactate levels between HC, IMa and IMb groups respectively over the first 5 h NMP, which included all 12 kidneys (2-way ANOVA, *p* = <0.031). Perfusate lactate levels were significantly lower in IMa compared with IMb but not HC versus IMa or IMb (2-way ANOVA with Tukey’s multiple comparisons over 5 h NMP, *p* = 0.028), Figure 3.

### Renal Function

**FIGURE 4:**
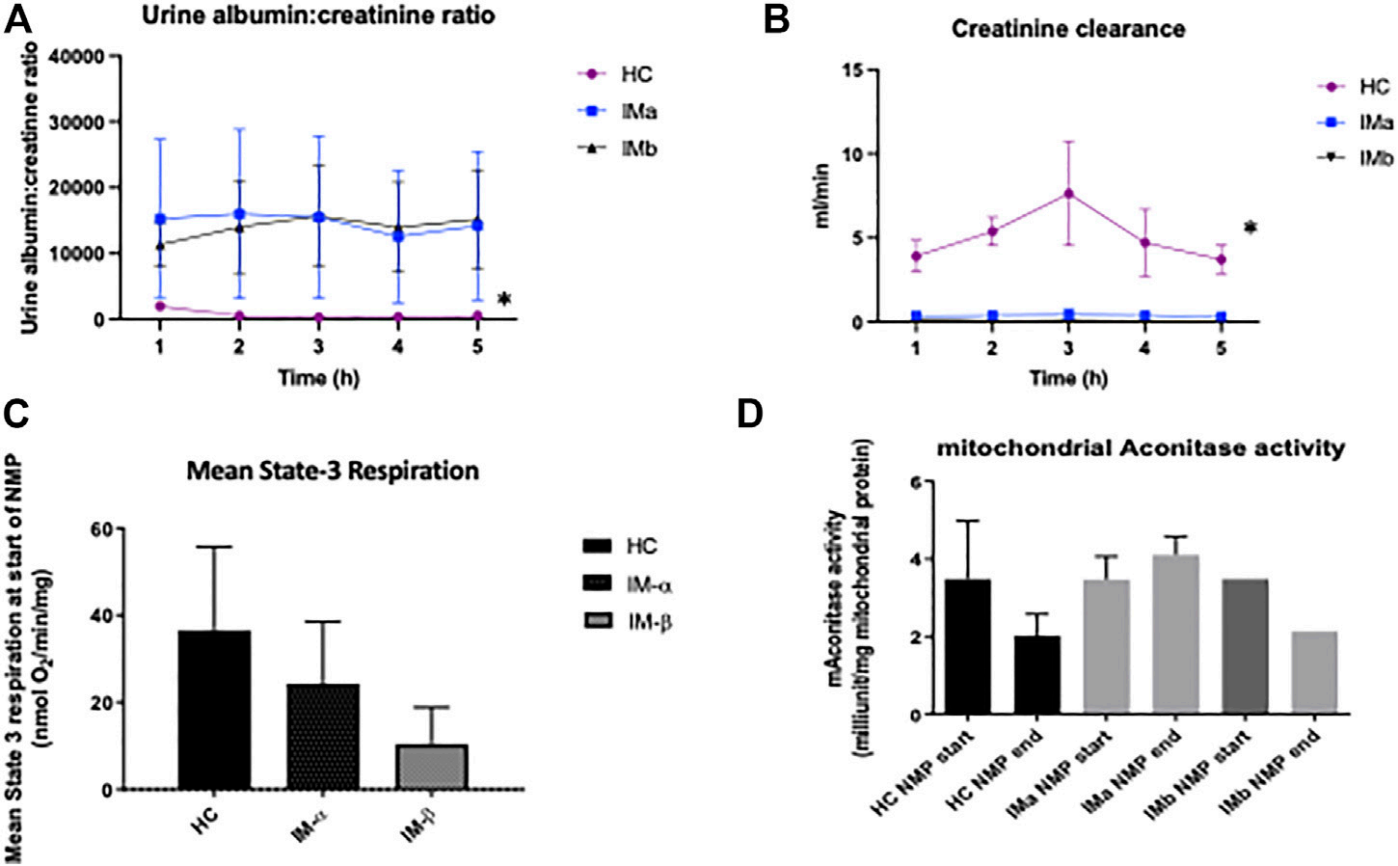
**(A)** Line graph showing urine albumin:creatinine ratio and **(B)** creatinine clearance during Normothermic Machine Perfusion of Healthy and Injury model kidney groups IMa and IMb (2-way ANOVA analysed over 5 h NMP with = *p* < 0.05 *). **(C)** Graph showing mean State-3 respiration and **(D)** mitochondrial aconitase activity during Normothermic Machine Perfusion of Healthy and Injury model kidney groups IMa and IMb (* = *p* < 0.05).

Creatinine clearance was significantly higher in the healthy control group compared to both IMa and IMb groups (Two-way ANOVA, *p* ≤ 0.0001) although there was no difference in CrCl over time (Two-way ANOVA with Tukey’s multiple comparisons over 5 h NMP, *p* = 0.88). Urine albumin:creatinine ratio was significantly lower in the healthy control group compared to both IMa and IMb groups (2-way ANOVA, *p* ≤ 0.0001) but with no difference over time (2-way ANOVA with Tukey’s multiple comparisons over 5 h NMP, *p* = 0.99), Figure 4.

### Markers of Injury

Perfusate LDH levels were significantly different between groups over 5 h NMP (AUC and One-way ANOVA: 2070 ± 952 vs. 4106 ± 319 vs. 3168 ± 700IU/L; *p* ≤ 0.02) with a significant difference between HC and IMa but not HC and IMb over 5 h NMP (AUC and One-way ANOVA with multiple comparisons (*p* < 0.05). Perfusate AST was no different over 5 h NMP (AUC and One-way ANOVA: 1089 ± 985 vs. 2447 ± 271 vs. 1757 ± 1189IU/L; *p* = 0.19), respectively, Figure 3.

### Mitochondrial Assessment

ADP-dependent (State-3) respiration is defined as ADP-dependent oxygen consumption, and reflects the mitochondrial respiration coupled with ATP production. This was measured “real-time” in mitochondria isolated from biopsies taken immediately prior to normothermic machine perfusion. A significant difference was seen between the 3 groups at the beginning of NMP (Kruskal-Wallis: 37 ± 19 vs. 24 ± 14 vs. 10 ± 8; nmolO_2_/min/mg; *p* = 0.049). Comparison between the groups showed a higher level of state-3 respiration in the HC group compared to IMb (Mann-Whitney, *p* = 0.0024) but not IMa (Mann-Whitney, *p* = 0.38). There was no difference in mean mitochondrial respiration throughout perfusion between the 3 groups (One-way ANOVA: 33 ± 23 vs. 29 ± 13 vs. 14 ± 7 nmolO_2_/min/mg; *p* = 0.38). Mitochondrial aconitase activity was measured as a marker of mitochondrial oxidative stress and there was no difference at the beginning and end of NMP in either healthy kidneys or injured kidneys and no difference between the two groups (One-way ANOVA *p* = 0.506), Figure 4.

### Histology

There was a significant difference in injury severity (ISS) score between the healthy control kidneys and both ischaemic model groups at the beginning of preservation (T0) (Chi-Squared: *p* = 0.01), however at the end of preservation (T8) there was no difference between HC and IM groups (Chi-squared *p* = 0.76). There was a significant worsening of injury severity score in HC from T0 to T8 (Chi-squared *p* = 0.007), but no difference in ISS in the IM kidneys from T0 to T8 (Chi-squared *p* = 0.99).

## Discussion

This study set out to assess whether mitochondrial respiratory capacity, analysed during normothermic machine perfusion (NMP), had the potential as an indicator of kidney viability. ADP-dependent state-3 respiration assessed from freshly isolated mitochondria at the end of the preservation period was lower in ischaemic kidneys that performed poorly during NMP (IMb), compared to healthy controls. This difference was significant despite the small numbers (*n* = 3) present in the two injury model groups. The original study design included paired kidneys to compare a “gold standard” healthy control with kidney injury that was at the extent of what would be considered viable. 60 min warm ischaemia with 24 h cold ischaemia was selected, as the combination of WI and CI was clinically relevant, and the durations were based on previous work that showed 75 min WI plus 16 h CI followed by auto-transplant led to significant but recoverable acute kidney injury [11]. During the project it became clear that in some of the kidneys the injury sustained was unrecoverable, as they were significantly deteriorating and appeared to be non-viable during NMP. We used pre-established criteria to decide upon perfusion termination (Supplementary Appendix SA1) and of the 6 injury group kidneys 3 were terminated early. This resulted in the injury group being split into two groups, those that completed 8 h of NMP successfully (IMa, *n* = 3) and those that were terminated early (IMb, *n* = 3). The authors recognise the limitation that the small group size has on the strength of the findings. With hindsight, it would have been beneficial to have continued the perfusion of all kidneys to the common endpoint of 8 h. However, the clear differences between the 2 sub-groups are discussed in detail below and to minimise the effect of the small group sizes we have mainly analysed the data over 5 h NMP which includes all 12 experimental kidneys.

The first interesting finding was the difference in perfusion parameters between the two injury groups during HMP. Kidneys from the injury model group that completed 8 h NMP successfully (IMa) had lower renal resistance at the beginning of HMP and higher renal blood flow at the beginning and end of HMP compared with kidneys in the injury model group that were terminated on NMP early (IMb). Despite these differences, the renal resistance significantly reduced in the IMb group throughout HMP and was no different to the IMa value at the end of HMP. This suggests that for IRR, the values obtained at the beginning of HMP may be more predictive of viability than the end values or the change during perfusion. Clinical studies have shown that renal resistance, both during HMP and at the end of perfusion may be predictive of long term graft survival [12, 13]. In a sub-group analysis of the European MP-trial a cut off of >0.4 mmHg/ml/min at the end of perfusion was suggested, which was based on HMP of human kidneys on the Lifeport Kidney Transporter [13]. Our study used pig kidneys on the Kidney Assist Transporter device and the IRR values at the end of perfusion were 0.25 (IMa group) and 0.51 (IMb group) which correlates with the >0.4 mmHg/ml/min, despite these clear methodological differences. Systematic review and meta-analysis data demonstrate that HMP of deceased donor kidneys reduces DGF but the effect on graft survival is less clear and there is no effect on PNF [14, 15]. As a predictor of function, there is not enough evidence that IRR during HMP can be used in isolation. However, it may be valuable in selecting those kidneys that are marginal and would benefit from additional testing such as NMP and/or biopsy.

ADP-dependent mitochondrial respiration was lower in the IMb group immediately prior to NMP compared with the HC and IMa groups. Despite the small numbers and variation at different time points throughout NMP, mitochondrial respiration remained stable across the groups, with similar values at the beginning and end of NMP. This suggests that 8 h NMP supports mitochondrial respiration but does not aid mitochondrial recovery. It is unclear whether this is due to the kidneys having sustained unrecoverable injury or whether the pseudo-physiological state during NMP does not provide the environment or substrates required. From the perspective of potential translation into organ assessment in the clinical field, it was interesting to observe that mitochondrial respiration showed the same pattern as calculated oxygen consumption. Calculated oxygen consumption can be assessed in real-time and requires only venous and arterial blood gas analysis. This is in contrast to isolating mitochondria and analysing fresh samples for respiratory function, which is very resource intensive and impractical in the clinical context. An alternative possibility would be to assess respiratory capacity directly in permeabilized tissue biopsies, avoiding the need for mitochondrial isolation, but unlike net oxygen consumption, allowing a thorough measurement of respiratory capacity [16]. In addition, the level of mitochondrial respiration at the beginning of NMP may have the potential to be predictive of organ viability. This study has not conclusively demonstrated a link between oxygen consumption/mitochondrial respiration and organ viability during NMP but it is clear that organs with poor perfusion parameters during NMP were those with low O_2_ consumption and low ADP-dependent state-3 respiration at the end of cold preservation.

**FIGURE 5:**
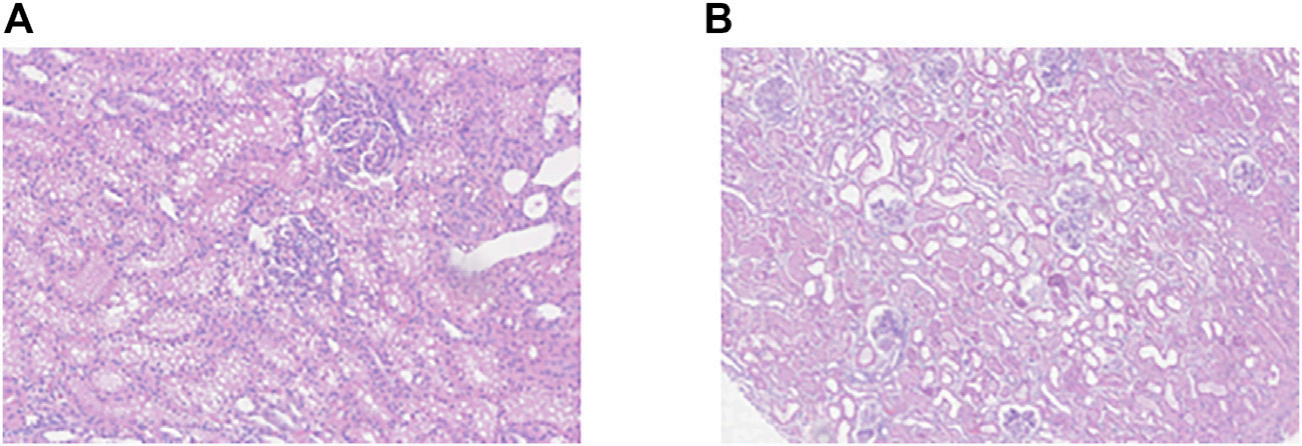
**(A)** Representative histological image of a healthy kidney section at 20x magnification after 8 h NMP showing significant cytoplasmic vacuolation. **(B)** histological image of an injury model kidney after 8 h NMP showing tubular dilatation.

Mitochondrial aconitase activity was measured as a marker of oxidative damage, as oxidative stress reversibly inactivates the enzyme. No significant differences were found between the groups, although interestingly the levels in HC kidneys dropped from the beginning to the end of perfusion. This suggests that NMP itself may cause oxidative damage, consistent with a reperfusion event after a period of sustained ischaemia [17] despite the lack of kidney injury. Histological data also show that NMP has an effect on healthy tissue, with an increase in the injury severity score (ISS). The ISS at the beginning of NMP showed, as expected, almost no injury, but after 8 h there was evidence of tubular injury and significant cytoplasmic vacuolation (Figure 5). However, the change in ISS in the IM groups during perfusion was not significant which makes histology difficult to interpret in the context of viability. A further interesting observation was that although the macroscopic appearance of the IMb kidneys was globally discoloured and ischaemic, the histology scores were no different from IMa and no different at the end of perfusion compared with the beginning. There was no cortical necrosis and tubular injury was mostly moderate, not severe. Other groups have shown that tubular injury and inflammation were lower in the 8 h NMP group 30 mins after auto-transplant compared to controls however the findings were marginal and did not persist at 3 days after transplant [18].

Healthy kidneys were deliberately selected as a control to determine the impact of NMP as a preservation technique on uninjured organs and to see what readings NMP of a “normal” kidney produced. Kidneys were perfused for 8 h and had stable blood flow and oxygen consumption throughout. Lactate levels remained stable, although cellular injury markers, AST and LDH did rise throughout perfusion. This pattern was also observed by Kaths et al in their DCD auto-transplant model [19]. Healthy kidneys in this study retained glomerular and tubular function, demonstrated by the ability to clear creatinine and the absence of proteinuria, which remained stable throughout the 8 h NMP. This was in contrast to kidneys in both injury groups which, despite the IMa group producing a good urine volume, did not clear creatinine and had gross proteinuria. This suggests that tubular and glomerular function during NMP may not be a helpful marker of viability in injured kidneys and could be considered the clinical equivalent of delayed graft function. Other groups have effectively used CrCl and fractional excretion of sodium during NMP to demonstrate function and compare kidneys although the kidney injury was less severe than in our model [20–22].

The striking feature of the IMa group that perfused successfully for 8 h was that absolute values of renal blood flow, oxygen consumption and urine output were higher than the healthy control group. In order to sustain ATP production, oxygen consumption increases which results in an increase in renal blood flow [23–25]. This results in an increased glomerular filtration rate, and hence increased urine output, as seen in the IMa group. Therefore, the tubular load of electrolytes destined for active reabsorption rises and the increased oxygen delivery is matched by increased demand. However, the inability of the kidney to compensate for increases in oxygen consumption renders it particularly sensitive to alterations in oxygen metabolism that result in decreased kidney oxygen tension (pO_2_) [26]. We did not directly measure tissue oxygen tension in this study but it is certainly worth further investigation and may be the most accurate way to assess this complex equilibrium during NMP and help decide on optimal pO_2_ value and oxygen delivery.

Although the objective of the study was the assessment of NMP as a preservation technique, the main limitation was the absence of a transplant end point, which offers clinically relevant functional data. In the context of animal ethics and refinement, we minimised the number of animals required, the cost and the severity of the model, without a significant compromise to the outcome [27]. A pig model was selected as the heterogeneity of discarded human kidneys makes mechanistic work challenging. The pig has genetic, anatomical, and immunological similarities to the human and is recognised as an excellent translational model [28–30]. The authors acknowledge that the concept of organ viability during NMP is difficult to define and for the kidneys that were considered non-viable (IMb group) and terminated on NMP early, they all scored 5 (data not shown) using the published viability score [3], which is considered un-transplantable. This score was designed for human kidneys undergoing 1 h of NMP and may not be directly translatable but is widely used in experimental work. We developed our own criteria for termination of pig kidney NMP on the basis of previous experience (Supplementary Appendix SA1). In addition, long term outcomes after DCD transplant are equivalent to DBD, despite the warm ischaemic injury the kidneys sustain. Other factors, such as chronic rejection, fibrosis and drug toxicity impact on graft survival and longevity, which are unlikely to be linked to biomarkers during NMP [31].

In conclusion, we have shown that this pig kidney model with 60 min warm ischaemia and 24 h cold ischaemia offers an extreme of injury that appears to be at the interface between viable and non-viable. We have shown the impact of NMP on uninjured, healthy kidneys as a benchmark for future work. We have demonstrated that ADP-dependent state-3 mitochondrial respiration levels at the beginning of NMP may be suggestive of kidney viability during NMP and that parameters directly measurable during NMP including oxygen consumption, renal blood flow and lactate can differentiate injured kidneys into those that are comparable with healthy controls and those that appear non-viable.

## Data Availability

The original contributions presented in the study are included in the article/Supplementary Material, further inquiries can be directed to the corresponding author.

## References

[1] ODT. NHSBT Transplant Activity Report 2018-2019 (2021). Available at: https://www.odt.nhs.uk/statistics-and-reports/annual-activity-report (Accessed November 17, 2021).

[2] Gueler F Gwinner W Schwarz A Haller H. Long-term Effects of Acute Ischemia and Reperfusion Injury. Kidney Int (2004) 66(2):523–7. 10.1111/j.1523-1755.2004.761_11.x 15253702

[3] Hosgood SA Barlow AD Hunter JP Nicholson ML. *Ex Vivo* normothermic Perfusion for Quality Assessment of Marginal Donor Kidney Transplants. Br J Surg (2015) 102(11):1433–40. 10.1002/bjs.9894 26313559

[4] Bagul A Hosgood SA Kaushik M Kay MD Waller HL Nicholson ML. Experimental Renal Preservation by Normothermic Resuscitation Perfusion with Autologous Blood. Br J Surg (2008) 95(1):111–8. 10.1002/bjs.5909 17696214

[5] Weissenbacher A Lo Faro L Boubriak O Soares MF Roberts IS Hunter JP Twenty‐four-hour Normothermic Perfusion of Discarded Human Kidneys with Urine Recirculation. Am J Transpl (2019) 19(1):178–92. 10.1111/ajt.14932 PMC6491986 29758129

[6] Wen-Xing D Xiao-Ming Y. Mitophagy: Mechanisms, Pathophysiological Roles, and Analysis. Biol Chem (2012) 393(7):547–64. 10.1515/hsz-2012-0119 22944659 PMC3630798

[7] Chouchani ET Pell VR James AM Work LM Saeb-Parsy K Frezza C A Unifying Mechanism for Mitochondrial Superoxide Production during Ischemia-Reperfusion Injury. Cell Metab (2016) 23(2):254–63. 10.1016/j.cmet.2015.12.009 26777689

[8] Schlegel A Muller X Mueller M Stepanova A Kron P de Rougemont O Hypothermic Oxygenated Perfusion Protects from Mitochondrial Injury before Liver Transplantation. EBioMedicine (2020) 60(10):103014. 10.1016/j.ebiom.2020.103014 32979838 PMC7519249

[9] Lohmann S Pool MBF Rozenberg KM Keller AK Moers C Møldrup U Mesenchymal Stromal Cell Treatment of Donor Kidneys during *Ex Vivo* Normothermic Machine Perfusion: A Porcine Renal Autotransplantation Study. Am J Transpl (2021) 21(7):2348–59. 10.1111/ajt.16473 33382194

[10] Frezza C Cipolat S Scorrano L. Organelle Isolation: Functional Mitochondria from Mouse Liver, Muscle and Cultured Filroblasts. Nat Protoc (2007) 2(2):287–95. 10.1038/nprot.2006.478 17406588

[11] Lohmann S Eijken M Møldrup U Møller BK Hunter J Moers C *Ex Vivo* Administration of Mesenchymal Stromal Cells in Kidney Grafts against Ischemia-Reperfusion Injury-Effective Delivery without Kidney Function Improvement Posttransplant. Transplantation (2021) 105(3):517–28. 10.1097/tp.0000000000003429 32956281

[12] Sandal S Paraskevas S Cantarovich M Baran D Chaudhury P Tchervenkov JI Renal Resistance Thresholds during Hypothermic Machine Perfusion and Transplantation Outcomes - A Retrospective Cohort Study. Transpl Int (2018) 31(6):658–69. 10.1111/tri.13146 29493843

[13] Jochmans I Moers C Smits JM Leuvenink HGD Treckmann J Paul A The Prognostic Value of Renal Resistance during Hypothermic Machine Perfusion of Deceased Donor Kidneys. Am J Transpl (2011) 11:2214–20. 10.1111/j.1600-6143.2011.03685.x 21834917

[14] Tingle SJ Figueiredo RS Moir JA Goodfellow M Talbot D Wilson CH. Machine Perfusion Preservation versus Static Cold Storage for Deceased Donor Kidney Transplantation. Cochrane Database Syst Rev (2019) 3(3):CD011671. 10.1002/14651858.CD011671.pub2 30875082 PMC6419919

[15] Peng P Ding Z He Y Zhang J Wang X Yang Z. Hypothermic Machine Perfusion versus Static Cold Storage in Deceased Donor Kidney Transplantation: A Systematic Review and Meta‐Analysis of Randomized Controlled Trials. Artif Organs (2019) 43(5):478–89. 10.1111/aor.13364 30282122

[16] Kuznetsov AV Strobl D Ruttmann E Königsrainer A Margreiter R Gnaiger E. Evaluation of Mitochondrial Respiratory Function in Small Biopsies of Liver. Anal Biochem (2002) 305(2):186–94. 10.1006/abio.2002.5658 12054447

[17] Chouchani ET Pell VR Gaude E Aksentijević D Sundier SY Robb EL Ischaemic Accumulation of Succinate Controls Reperfusion Injury through Mitochondrial ROS. Nature (2014) 515(7527):431–5. 10.1038/nature13909 25383517 PMC4255242

[18] Hamar M Urbanellis P Kaths MJ Kollmann D Linares I Ganesh S Normothermic *Ex Vivo* Kidney Perfusion Reduces Warm Ischemic Injury of Porcine Kidney Grafts Retrieved after Circulatory Death. Transplantation (2018) 102(8):1262–70. 10.1097/tp.0000000000002245 29683999

[19] Kaths JM Cen JY Chun YM Echeverri J Linares I Ganesh S Continuous Normothermic *Ex Vivo* Kidney Perfusion is Superior to Brief Normothermic Perfusion Following Static Cold Storage in Donation after Circulatory Death Pig Kidney Transplantation. Am J Transpl (2017) 17(4):957–69. 10.1111/ajt.14059 27647696

[20] Adams TD Hosgood SA Nicholson ML. Physiological Effects of Altering Oxygenation during Kidney Normothermic Machine Perfusion. Am J Physiol Renal Physiol (2019) 316(5):F823–F829. 10.1152/ajprenal.00178.2018 30785351

[21] Hosgood SA Moore T Qurashi M Adams T Nicholson ML. Hydrogen Gas Does Not Ameliorate Renal Ischemia Reperfusion Injury in a Preclinical Model. Artif Organs (2018) 42(7):723–7. 10.1111/aor.13118 29611214

[22] Smith SF Adams T Hosgood SA Nicholson ML. The Administration of Argon during *Ex Vivo* Normothermic Perfusion in an Experimental Model of Kidney Ischemia-Reperfusion Injury. J Surg Res (2017) 218:202–8. 10.1016/j.jss.2017.05.041 28985850

[23] Papazova DA Friederich-Persson M Joles JA Verhaar MC. Renal Transplantation Induces Mitochondrial Uncoupling, Increased Kidney Oxygen Consumption, and Decreased Kidney Oxygen Tension. Am J Physiol Renal Physiol (2015) 308(1):F22–F28. 10.1152/ajprenal.00278.2014 25275014

[24] Plotnikov EY Kazachenko AV Vyssokikh MY Vasileva AK Tcvirkun DV Isaev NK The Role of Mitochondria in Oxidative and Nitrosative Stress during Ischemia/reperfusion in the Rat Kidney. Kidney Int (2007) 72:1493–502. 10.1038/sj.ki.5002568 17914353

[25] Echtay KS Roussel D St-Pierre J Jekabsons MB Cadenas S Stuart JA Superoxide Activates Mitochondrial Uncoupling Proteins. Nature (2002) 415:96–9. 10.1038/415096a 11780125

[26] Oostendorp M de Vries EE Slenter JMGM Peutz-Kootstra CJ Snoeijs MG Post MJ MRI of Renal Oxygenation and Function after Normothermic Ischemia-Reperfusion Injury. NMR Biomed (2011) 24:194–200. 10.1002/nbm.1572 20954164

[27] Prescott MJ Lidster K. Improving Quality of Science through Better Animal Welfare: The NC3Rs Strategy. Lab Anim (2017) 46:152–6. 10.1038/laban.1217 28328893

[28] Baehr A Klymiuk N Kupatt C. Evaluating Novel Targets of Ischemia Reperfusion Injury in Pig Models. Int J Mol Sci (2019) 20, 4749. 10.3390/ijms20194749 PMC6801853 31557793

[29] Dondelinger RF Ghysels MP Brisbois D Donkers E Snaps FR Saunders J Relevant Radiological Anatomy of the Pig as a Training Model in Interventional Radiology. Eur Radiol (1998) 8:1254–73. 10.1007/s003300050545 9724449

[30] Dawson HD Smith AD Chen C Urban JF. An In-Depth Comparison of the Porcine, Murine and Human Inflammasomes; Lessons from the Porcine Genome and Transcriptome. Vet Microbiol (2017) 202:2–15. 10.1016/j.vetmic.2016.05.013 27321134

[31] Summers DM Johnson RJ Hudson A Collett D Watson CJ Bradley JA. Effect of Donor Age and Cold Storage Time on Outcome in Recipients of Kidneys Donated after Circulatory Death in the UK: A Cohort Study. Lancet (2013) 381(9868):727–34. 10.1016/s0140-6736(12)61685-7 23261146

