# Supplementary material for: Assessment of Mitochondrial Function and Oxygen Consumption Measured During Ex Vivo Normothermic Machine Perfusion of Injured Pig Kidneys Helps to Monitor Organ Viability
Source: Transpl Int. 2022 May 31;35:10420. doi: 10.3389/ti.2022.10420 (PMC9194576; doi:10.3389/ti.2022.10420)
Supplement: Supplementary file 1 [file DataSheet1.docx]

**Appendix 1.**

Termination of the kidney perfusion experiment occurs when macroscopic perfusion is globally poor with at least of two the following criteria:

1.      Perfusate Flow <50 ml/min/100g

2.      Perfusate pH <7 or pH >7.7 measured at a pCO_2_=5KPa

3.      Perfusate pCO_2_ levels > 6.5KPa

**Appendix 2**

Assessment of mitochondrial function in an IRI model

In previous experiments we were able to analyse different aspects of mitochondrial function in a rodent model of unilateral ischaemia-reperfusion injury in the kidney. In the model, the animals (adult male Fisher rats) were anesthetised with isoflurane and placed on an auto-thermoregulatory heating pad. Continuous monitoring of oxygen saturation and heart rate was performed throughout surgery. Following midline laparotomy, dissection of both the right and left renal pedicles was performed. The left renal pedicle was then clamped for 45 minutes, following administration of heparin (10 units/ml), while the right kidney remained untouched. Ischaemia was confirmed by visual inspection. After 45 minutes the clamp was removed and reperfusion observed. The animals were then recovered and reperfusion injury was assessed after 24h. The left kidneys were compared to the endogenous right kidney controls and to kidneys from healthy (sham operated) rats.

Mitochondrial function was assessed by investigating O_2_ consumption (using a Clark-type electrode) and enzymatic complex activities in isolated mitochondria and ATP levels in tissue.

Mitochondrial O_2_ consumption

**Figure 1:** Mitochondria isolated from IRI kidneys presented significantly lower O_2_ consumption (represented as respiratory control ratio, RCR) compared to endogenous and healthy controls. Data were analysed by 1-way ANOVA, correcting for multiple comparisons, *** P-value =0.0002

In our study we will only compare the means of two groups of kidneys (NMP vs sCS) and based on the above findings we can perform the following sample size calculation (software G*Power 3.9.1.2, Faul et. Al, Behavior Research Methods 2009, 41 (4), 1149-1160).

t tests - Means: Difference between two independent means (two groups)

Analysis A priori: Compute required sample size

Input: Tail(s) = Two

Effect size d= 7.1434904 (based on rodent experiments findings)

α err prob = 0.05

Power (1-β err prob) = 0.95

Allocation ratio N2/N1= 1

Output: Noncentrality parameter δ= 8.7489532

Critical t= 2.7764451

Df= 4

Sample size group 1= 3

Sample size group 2= 3

Total sample size= 6

Actual power= 0.9999834

N=3 kidneys per group should allow us to detect significant differences (p=0.05) in RCRs, with 95% power.

ATP levels

**Figure 2:** IRI left kidneys presented decreased ATP levels compared to healthy and endogenous controls, as measured by chemiluminescence (Enliten, Promega). Kruskal-Wallis test corrected for multiple comparisons, *p<0.05

Based on the findings from Figure 2 we can perform the following sample size calculation (software G*Power 3.9.1.2, Faul et. Al, Behavior Research Methods 2009, 41 (4), 1149-1160).

t tests - Means: Wilcoxon-Mann-Whitney test (two groups) (non-normally distributed data)

Options: A.R.E. method

Analysis A priori: Compute required sample size

Input: Tail(s) = Two

Parent distribution= Normal

Effect size d= 1.6315529 (based on rodent experiments findings)

α err prob= 0.05

Power (1-β err prob) = 0.80

Allocation ratio N2/N1 = 1

Output: Noncentrality parameter δ= 3.1887234

Critical t= 2.1557656

Df= 13.2788745

Sample size group 1= 8

Sample size group 2= 8

Total sample size= 16

Actual power= 0.8392710

N=8 kidneys per group should allow us to detect significant differences (p=0.05) in ATP levels, with 80-84% power.

Complex I activity

**Figure 3:** Mitochondria isolated from IRI left kidneys presented decreased complex I activity compared to healthy and endogenous controls. Unpaired t test, LK vs RK or LK vs HC. *p=0.0227, **p=0.0028

Based on the findings from Figure 3 we can perform the following sample size calculation (software G*Power 3.9.1.2, Faul et. Al, Behavior Research Methods 2009, 41 (4), 1149-1160).

t tests - Means: Difference between two independent means (two groups)

Analysis A priori: Compute required sample size

Input: Tail(s) = Two

Effect size d= 1.7790919

α err prob= 0.05

Power (1-β err prob) = 0.80

Allocation ratio N2/N1 = 1

Output: Noncentrality parameter δ= 3.3283762

Critical t= 2.1788128

Df= 12

Sample size group 1= 7

Sample size group 2= 7

Total sample size= 14

Actual power= 0.8627720

N=7 kidneys per group should allow us to detect significant differences (p=0.05) in Complex I activity, with 80-86% power.
